# TLR7 agonist, DSP-0509, with radiation combination therapy enhances anti-tumor activity and modulates T cell dependent immune activation

**DOI:** 10.1186/s12865-024-00643-x

**Published:** 2024-07-25

**Authors:** Yosuke Ota, Ryosaku Inagaki, Yasuhiro Nagai, Yuko Hirose, Masashi Murata, Setsuko Yamamoto

**Affiliations:** Cancer Research Unit, Sumitomo Pharma Co Ltd, Osaka, Japan

**Keywords:** TLR7 agonist, DSP-0509, Immunoradiotherapy, Predictive marker

## Abstract

**Background:**

TLR7 is a key player in the antiviral immunity. TLR7 signaling activates antigen-presenting cells including DCs and macrophages. This activation results in the adaptive immunity including T cells and B cells. Therefore, TLR7 is an important molecule of the immune system. Based on these observations, TLR7 agonists considered to become a therapy weaponize the immune system against cancer. Radiation therapy (RT) is one of the standard cancer therapies and is reported to modulate the tumor immune response. In this study, we aimed to investigate the anti-tumor activity in combination of TLR7 agonist, DSP-0509, with RT and underlying mechanism.

**Result:**

We showed that anti-tumor activity is enhanced by combining RT with the TLR7 agonist DSP-0509 in the CT26, LM8, and 4T1 inoculated mice models. We found that once- weekly (q1w) dosing of DSP-0509 rather than biweekly (q2w) dosing is needed to achieve superior anti-tumor activities in CT26 model. Spleen cells from the mice in RT/DSP-0509 combination treatment group showed increased tumor lytic activity, inversely correlated with tumor volume, as measured by the chromium-release cytotoxicity assay. We also found the level of cytotoxic T lymphocytes (CTLs) increased in the spleens of completely cured mice. When the mice completely cured by combination therapy were re-challenged with CT26 cells, all mice rejected CT26 cells but accepted Renca cells. This rejection was not observed with CD8 depletion. Furthermore, levels of splenic effector memory CD8 T cells were increased in the combination therapy group. To explore the factors responsible for complete cure by combination therapy, we analyzed peripheral blood leukocytes (PBLs) mRNA from completely cured mice. We found that Havcr2^low^, Cd274^low^, Cd80^high^, and Il6^low^ were a predictive signature for the complete response to combination therapy. An analysis of tumor-derived mRNA showed that combination of RT and DSP-0509 strongly increased the expression of anti-tumor effector molecules including Gzmb and Il12.

**Conclusion:**

These data suggest that TLR7 agonist, DSP-0509, can be a promising concomitant when used in combination with RT by upregulating CTLs activity and gene expression of effector molecules. This combination can be an expecting new radio-immunotherapeutic strategy in clinical trials.

**Supplementary Information:**

The online version contains supplementary material available at 10.1186/s12865-024-00643-x.

## Background

In recent years, the advent of anti-PD-1 / PD-L1 antibodies in cancer therapy has led to the development of immunotherapy as a standard of care, but its efficacy is limited to a subset of patients, therefore novel therapies are required. TLRs function in the early innate immune response and play an essential role in immune system activation, especially in response to infectious diseases [1, 2]. Among TLRs, TLR7 is expressed in intracellular endosome of DCs, macrophages, and B cells [3, 4]. TLR7 activates inflammatory signaling by activating transcription factors including NF-κB and IRF7 through MYD88 in response to ligands. These finally result in enhancing cytokine secretion and antigen presentation [5, 6]. Thus, TLR7 agonists are promising candidates for anti-tumor immune therapy and imiquimod is indeed an approved treatment for basal cell carcinoma [7, 8]. In addition, resiquimod of TLR7 agonist or PF‐3512676 of TLR9 agonist were reported to exhibit significant anti-tumor activity in cutaneous lymphomas [9, 10]. Because most of TLR agonists are restricted to apply topical use due to the physicochemical property including low solubility, developing a novel systemically administrable TLR7 agonist is expected. Based on this demand, multiple TLR7 agonists are developed in clinical trial [11]. Radiation therapy (RT) is widely used as a standard therapy for cancer treatment. One of the advantages in RT is the direct cytocidal action of irradiation can be differentiated from the systemic side effects. Fractionated irradiation allows the recovery of normal cells from sub-lethal damage between radiation schedules, which provides enough time to repopulate the normal cells [12]. Therefore, this approach has been applied clinically. It recently became revealed that the immunostimulatory effect depends on the irradiation method [13]. In addition to the cytocidal effect, attention has been given to the immunostimulatory effects of RT, which are attributable to mechanisms such as the abscopal effect and immunogenic cell death (ICD) in the tumor microenvironment [13]. It is reported that HMGB1, calreticulin, MHC class I, and induction of cytokines have roles in the mechanism of RT-induced ICD [14]. Based on these reports, combinations of RT with immunotherapy to maximize anti-tumor immunity are under investigation [15]. On the other hand, RT has also been reported to lead to increase immunosuppressive cells such as tumor-associated macrophages (TAMs), myeloid-derived suppressor cells (MDSCs), and regulatory T cells (Tregs) in the tumor microenvironment [16–18]. Therefore, details of the optimal regimen including dose and schedule of the RT and type of immunotherapy combined with RT are important to maximize anti-tumor immunity [15]. Although many researchers have conducted clinical trials of RT combination therapy with anti-PD-1 antibodies or anti-CTLA-4 antibodies, they have come to no definitive conclusions so far [19]. We have shown that DSP-0509, a novel systemically administrable TLR7 selective agonist, reduce the systemic immune side effects of TLR7 by shortening its in vivo half-life with maintaining its strong immune activity [20]. We have reported previously that DSP-0509 activated CD8 cells working in conjunction with anti-PD-1 antibodies and other immune checkpoint blockers (ICBs) in the tumor microenvironment and resulted in additive/synergistic anti-tumor activity [20]. Then, DSP-0509 was evaluated in clinical trial (NCT03416335). In the present study, we assessed the anti-tumor activity of RT combined with systemic administered DSP-0509. In addition, we showed that cancer-antigen recognizing CTLs result in prolonged survival in a mouse model. Furthermore, we found a specific gene signature in the peripheral blood to predict complete regression of tumor after treatment with combined RT and DSP-0509. Finally, we showed that gene expression related to CD8 T cells, NK cells, and DCs were increased in the tumor microenvironment leading to potent anti-tumor activity.

## Methods

### Compounds and dosing solutions

DSP-0509 was chemically synthesized at Sumitomo Pharma Co., Ltd. For in vitro studies, 10 mM solutions were prepared by dissolution in DMSO followed by dilution of the DMSO up to a final concentration of 0.1%. For in vivo studies, the compounds were dissolved in 2.5 mM glycine buffer solution of pH 10.2.

### Cells and cultures

CT26 and 4T1 cell lines were obtained from the American Type Culture Collection (ATCC). LM8 cell line was obtained from RIKEN Bio Resource Research Center (RIKEN BRC). Renca cell line was kindly provided by Dr. Fujioka, Iwate Medical University School of Medicine. CT26, 4T1, and Renca cells were maintained in culture by passage 1–2 times a week in RPMI 1640 supplemented with 10% FBS, penicillin/streptomycin. LM8 was maintained in culture by subculturing cells 1–2 times a week in MEM supplemented with 10% FBS, penicillin/streptomycin.

### Mice

We used 6- to 10-week-old female Balb/c mice purchased from Charles River Japan or CLEA Japan and 6- to 10-week-old female C3H/HeN mice purchased from Charles River Japan. Mice were fed a CE-2 normal diet (CLEA Japan). The animal room was maintained at 20–26 °C and humidity at 40–70%. The animal room lights were lit on a 12-hour cycle. After the study, mice were euthanized with CO_2_ inhalation. All animal studies were conducted in compliance with the Sumitomo Pharma Animal Ethics Code.

### In vivo anti-tumor study

CT26, 4T1, and Renca cells were suspended in HBSS and implanted subcutaneously into Balb/c mice. LM8 cells were implanted into C3H/HeN mice. The number of implanted cells per mouse was 1 × 10^6^ for CT26 and 4T1, 1 × 10^5^ for Renca, and 2.5 × 10^6^ for LM8. All implantation was conducted under anesthesia with isoflurane at 2.5 L/min. When the tumors reached approximately 100 mm^3^, the mice were randomized into groups. Bolus i.v. DSP-0509 was administered at 5 mg/kg in all studies. Dosing interval was once a week for DSP-0509 unless otherwise described in the figure legend. A dose of ionizing radiation was delivered to tumors on 5 sequential days starting from the first day of DSP-0509 dosing. Mice were anesthetized with isoflurane at 2.5 L/min during radiation and all non-tumor bearing parts of the mouse were shielded with a lead plate. An M-150WE X-ray system was used to generate the radiation (SOFTEX). For the re-challenge study, 1 × 10^6^ CT26 or Renca cells were inoculated into the dorsal flanks of completely cured or naïve mice. To deplete CD8 T cells, 200 µg of anti-CD8 antibody (BioXcell, clone 2.43) or 200 µg of rat IgG2b isotype antibody (BioXcell, clone LTF-2) was administered once every other day for 3 times. In the CD8 depletion study, CT26 was implanted at the day of the second dose of antibody. The tumor volume was calculated using the formula (L × W^2^) / 2, where L and W refer to the length and width dimensions, respectively.

### qRT-PCR

Total RNA was isolated from peripheral blood cells or in vivo implanted tumors. Tumors were snap frozen on dry ice upon collection. Blood was hemolyzed and total RNA was isolated from leukocytes using the QIAamp RNA Blood Mini Kit (Qiagen). Tumors were homogenized and total RNA was isolated using the RNeasy Mini Kit (Qiagen). The resulting total RNA was subjected to reverse transcription using a High-Capacity cDNA Reverse Transcription Kit (ThermoFisher) to synthesize cDNA. The synthesized cDNA was amplified in a quantitative PCR (qPCR) containing SsoFast EvaGreen Supermix with low ROX (BioRad) and analyzed using the Biomark HD system (Fluidigm). The primers for the qPCR used in this study are listed in Supplementary Table 2. Raw data was listed in Supplementary Table 3.

### Chromium release assay

Spleen cells were collected 24 h after the third administration of DSP-0509. Spleen cells were incubated for 5 days in the presence of 1 µg/mL H-2Ld MuLV gp70 peptide (MBL). Target CT26 cells were labeled with sodium ^51^Cr for 2 hours before coculturing with effector spleen cells. Five hundred labeled CT26 cells were incubated for 6 h with effector spleen cells in the ratio of 1:80, 1:40, 1:20, and 1:10, respectively. Radioactivity in the collected supernatants was measured using a 2470 WIZARD^2^ gamma counter (Perkin Elmer). Total (100%) cell lysis was defined as the radioactivity of CT26 cells completely lysed by the addition of NP40. No (0%) cell lysis was defined as the radioactivity of labeled CT26 cells without effector cells.

### ELISPOT assay

The mouse IFNγ ELISPOT set (BD Biosciences) was used for the ELISPOT assay. Spleens were isolated from tumor-bearing or naïve mice. Isolated spleen cells were stimulated in the presence of 1 µg/mL H-2Ld MuLV gp70 peptide (MBL) for 5 days. In the ELISPOT assay, 5 × 10^5^ stimulated spleen cells were incubated with or without 50 µg/mL H-2Ld MuLV gp70 peptide for 16 h on polyvinylidene fluoride (PVDF) plates. The number of IFNγ-positive cells was counted and analyzed by Immuno Spot Analyzers (CTL).

### Flow cytometry analysis

Flow cytometry analysis was carried out using the following antibodies: FITC-CD8a (BD 53 − 6.7), PerCP-Cy5.5-CD62L (eBioscience, MEL-14), PE-Cy7-CD127 (eBioscience, A7R34), FITC-CD3ε (BD Bioscience, 145-2C11), PE-CD4 (BD Bioscience, H129.19), APC-CD8a (BD Bioscience, 53 − 6.7), and V450-CD45 (BD Bioscience, 30-F11). Fixable Viability Stain 450 (BD Horizon) was used to discriminate live from dead cells. To analyze the flow cytometry data, FlowJo (BD Biosciences) was used. The effector memory T cell population was analyzed by gating the CD62L^−^CD127^+^ population of CD8^+^ T cells after gating out FVS 450-positive dead cells and gating the lymphocyte population in the FSC/SCC plot.

### Data and statistical analysis

Stat Preclinica Client (SAS 9.4, Takumi Information Technology Inc.) was used for statistical analysis in the in vivo anti-tumor studies. To compare differences between the two groups, unpaired two-tailed t-tests were conducted. In the in vivo tumor study, one-way repeated ANOVA was used, followed by post hoc Dunnett or Tukey tests. In Kaplan Meier survival analysis, a log rank test was used for comparison. All data are presented as mean ± SEM. Analysis of qPCR data was done using R statistical software (v4.1.2). Delta threshold cycle (dCt) values were calculated by subtracting Ct values of a housekeeping gene (PPIA) from Ct values of each gene. Subsequent analysis was done using dCt values. Principal component analysis (PCA), hierarchical clustering, and receiver operating characteristic (ROC) analysis were performed using the prcomp function of the stats package, pheatmap function of the pheatmap package, and roc function of the pROC package, respectively. The multi-gene logistic regression models for predicting probability to achieve CR were built using the glm function and backward feature selection (step function), in which the top 8 genes in terms of AUC in a single gene ROC analysis were used as the starting feature set. Differentially expressed genes for each dosing group versus the vehicle group were selected by the Student *t*-test and visualized by using the ggVennDiagram library.

## Results

### Systemic DSP-0509 enhanced the anti-tumor activity of RT in the CT26 tumor-bearing model

**Fig. 1:**
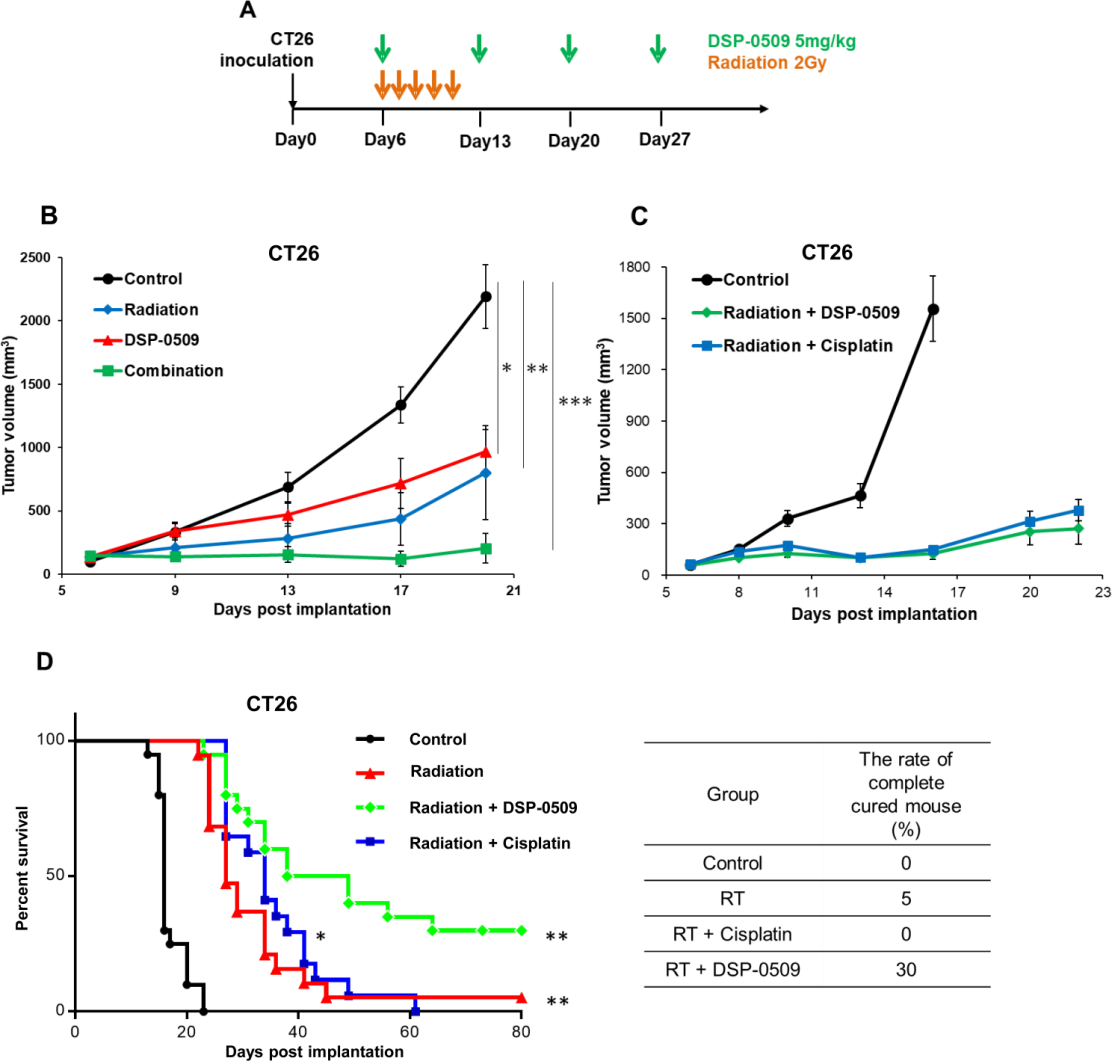
Systemic DSP-0509 enhanced the anti-tumor activity of RT in the CT26 tumor-bearing model. **A.** Study schedule for RT combined with DSP-0509 in the CT26 model. **B.** Tumor growth curve in the CT26 model. DSP-0509 was administered at 5 mg/kg q1w intravenously. Radiation was dosed at 2 Gy/day for five sequential days from the starting day of DSP-0509 dosing. *n* = 6 / group. * *P* < 0.05, **P < 0.01, ***P < 0.001 vs. control with the Dunnett test. The values are the mean and SEM (error bar). **C**. Curve of tumor growth in the CT26 model. DSP-0509 was administered at 5 mg/kg q1w intravenously. Cisplatin was administered 5 mg/kg q1w. *n* = 10 / group. **D.** Curve of Kaplan-Meier survival in the CT26 tumor-bearing model. End of survival was defined by tumor volume exceeding 1000 cm^3^

We evaluated the anti-tumor activity of RT with DSP-0509 in subcutaneously implanted CT26 tumor-bearing mice. DSP-0509 was administered intravenously at 5 mg/kg weekly and RT with 2 Gy was administered for 5 consecutive days starting from the day of the first dose of DSP-0509. The dosing schedule followed a previous report in which the combination of a TLR7 agonist and RT (Fig. 1A) [21, 22] were assessed. In the DSP-0509 monotherapy group and radiation monotherapy group, tumor growth inhibitory activity was significant compared to control group. Furthermore, tumor growth inhibitory activity was enhanced in the combination group (Fig. 1B). In addition, the combination of DSP-0509 and radiation did not reduce body weight (Supplementary Fig. 1A). To explore the difference between chemoradiation and immunoradiation therapy, we compared the anti-tumor activity by DSP-0509 combination and cisplatin combination. Tumor growth inhibition in combination of DSP-0509 with RT was not different from that of combination of cisplatin with RT at day 22 (Fig. 1C). Interestingly, the tumors were completely cured in 30% of the tumor-bearing mice of the DSP-0509 combination group, whereas none of the mice in the cisplatin combination group eradicated the tumors (Fig. 1D). Furthermore, body weight reduction in DSP-0509 combination group was less than that of in cisplatin combination group (Supplementary Fig. 1B). These results demonstrated that DSP-0509 in combination with RT enhanced tumor growth inhibition in the CT26 tumor-bearing murine model when compared to mice treated with RT alone. Furthermore, we found that the combination of RT with DSP-0509 led to enhanced long-term survival when compared with the combination of cisplatin with RT without reducing body weight.

### Anti-tumor activity was enhanced by the combination of DSP-0509 with RT in the 4T1 model

**Fig. 2:**
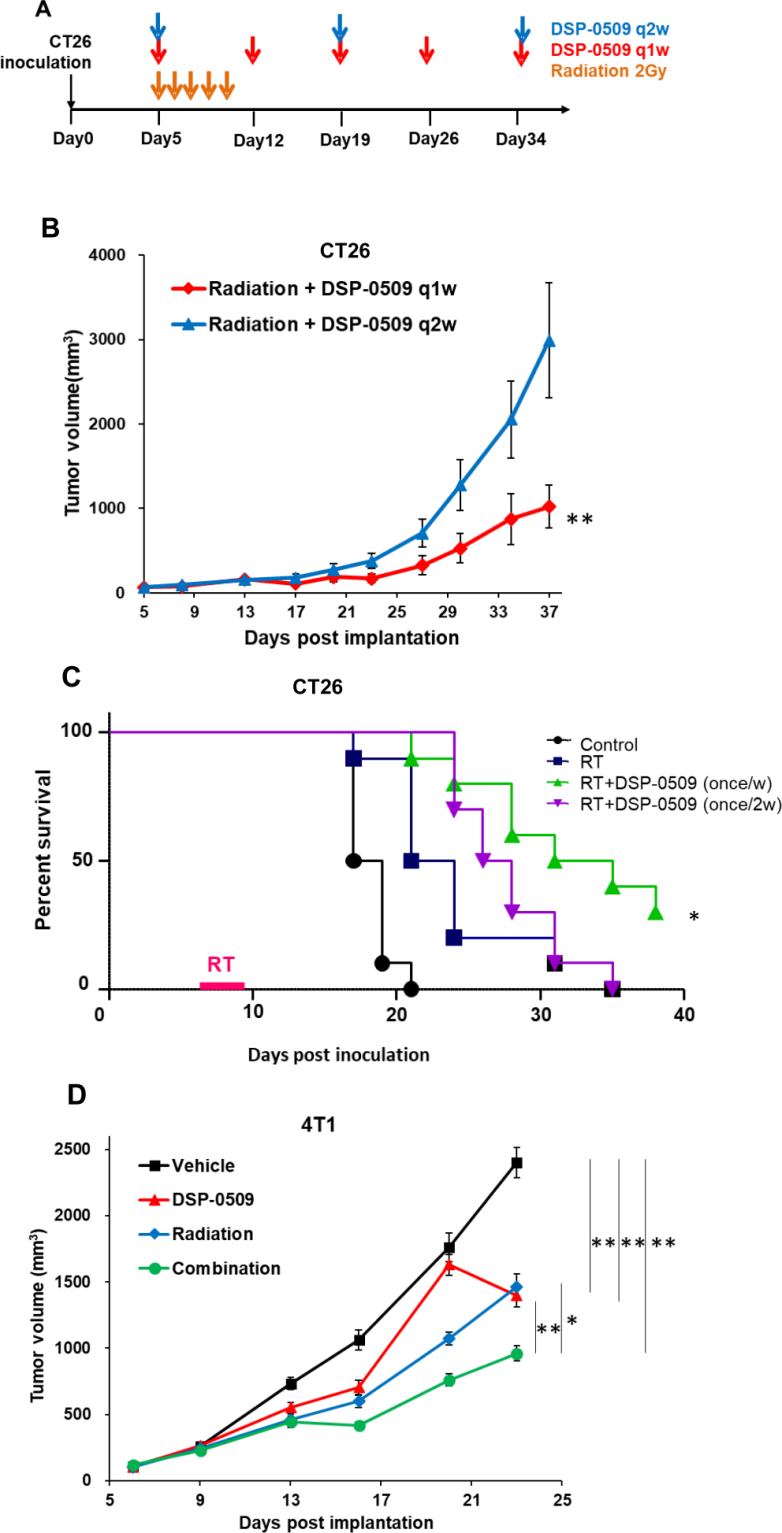
Dosing schedule affected the systemic DSP-0509-enhanced anti-tumor activity of RT in the 4T1 model. **(A)** Study schedule for RT combined with DSP-0509 in the CT26 model. **(B)** Anti-tumor activity in CT26 cells with different dosing schedules of DSP-0509. *n* = 10 / group and **P < 0.01 by the Dunnett test. **(C)** Kaplan-Meier curve of the data in Fig. 2B. *P < 0.05 Log-rank test vs. q2w group. **(D)** The 4T1 tumor growth curve. DSP-0509 was administered at 5 mg/kg once a week intravenously. Radiation was dosed at 2 Gy/day for five sequential days from the starting day of DSP-0509 dosing. **P < 0.01, *P < 0.05 for the Tukey test. *n* = 8 / group and the values are the mean and SEM (error bar)

As dosing intervals of TLR7 agonists have been reported to be an important factor for immunostimulatory activity [23], we compared the effect of once weekly (q1w) DSP-0509 dosing frequency with that of biweekly (q2w) on the anti-tumor activity of the RT and DSP-0509 combination (Fig. 2A). As a result, the q1w schedule was associated with stronger tumor growth inhibitory activity compared to the q2w schedule (Fig. 2B and Supplementary Fig. 2A). Furthermore, in CT26 tumor-bearing mice, survival was significantly prolonged when DSP-0509 was administered on a q1w schedule in combination with RT rather than on a q2w schedule in combination with RT (Fig. 2C). Next, to confirm that enhancement of anti-tumor activity by combined RT and DSP-0509 in CT26 mice could also be observed in other tumor-bearing mouse models, we evaluated the anti-tumor activity of this combination in the subcutaneous 4T1 tumor-bearing murine model. Consequently, when compared to the control, RT or DSP-0509 monotherapy showed statistically significant tumor growth inhibition, whereas compared to each monotherapy, the combination caused significant tumor growth inhibition (Fig. 2D). In addition, since the enhancement of the anti-tumor effect by combination of RT with TLR7 agonist was reported to be dependent on the irradiation dose [21, 22], we evaluated the anti-tumor activity of DSP-0509 (5 mg/kg q1w i.v.) combined with RT (2 Gy x 5 days) or RT (7 Gy x 5 days) in the subcutaneous LM8 tumor-bearing mouse model. Although there was a slight anti-tumor effect in the 2 Gy RT group, it was not statistically significantly different from that in the control group. On the other hand, when compared to the control, the group receiving combined 2 Gy RT and DSP-0509 manifested statistically significant tumor growth inhibition (Supplementary Fig. 3A). Although RT with 7 Gy also resulted in significant tumor growth inhibition, the combination of DSP-0509 with 7 Gy RT did not further increase the anti-tumor activity (Supplementary Fig. 3A). The survival of LM8 tumor-bearing mice treated with DSP-0509 combined with 2 Gy RT was significantly prolonged when compared to the control group (Supplementary Fig. 3B). These results suggested that the enhancement of the anti-tumor effect of RT combined with DSP-0509 can be observed in multiple models although the magnitude of the augmentation was different depending on models.

### Combination of RT with DSP-0509 induced anti-tumor CTLs and memory T cells

**Fig. 3:**
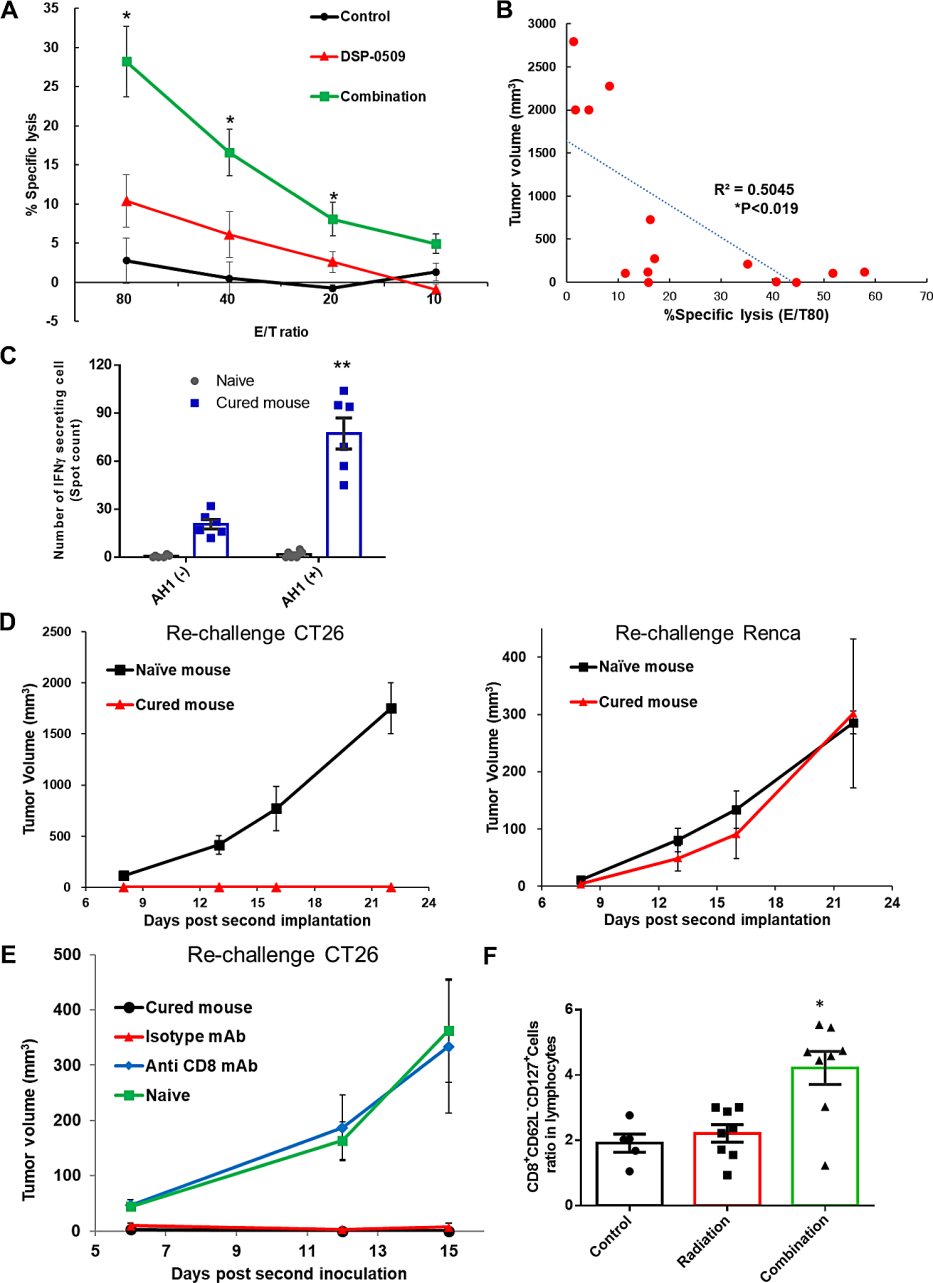
Combination of RT with DSP-0509 induced anti-tumor antigen CTLs and memory T cells. **(A)** Cytotoxicity assessment by Chromium release assay. Spleen cells from each group were incubated with AH1 peptide followed by ^51^Cr labeled CT26 cells. *n* = 3–6 in each group. *P < 0.05 for the Dunnett test. The values are the mean and S.E.M. (error bar). **(B)** Correlation between tumor volume at day 30 and cytotoxicity (Fig. 3A). *P < 0.05 calculated by a test for no correlation. R means Pearson’s correlation coefficient. **(C)** The results of the ELISPOT assay. Spleen cells from completely cured or naïve mice were stimulated with or incubated without AH1 peptide. *n* = 6 / group and **P < 0.01 vs. AH1(-) by a two-sided Student *t*-test. **(D)** Tumor growth in cured or naïve mice after re-challenge with CT26 cells. CT26 cells were inoculated on the right side and Renca cells were inoculated on the left side. *n* = 5 for each group and error bars show the SEM. **(E)** Tumor growth in completely cured or naïve mice after re-challenge. CD8 T cells was depleted before re-challenge with CT26 cells. *n* = 5 for each group and error bars show the SEM. **(F)** Effector memory T cells in peripheral blood were analyzed by flow cytometry. Effector memory T cells are defined as CD8^+^CD127^+^CD62L^−^. PBMCs were collected 7 days after the third dose of DSP-0509. Values are the mean ± SEM of each group. *n* = 5–8 / group. *P < 0.01 by the parametric the Dunnett test vs. the control group

To investigate the involvement of RT with DSP-0509-induced CTLs in tumor elimination, the tumoricidal activity of CTLs in splenocytes was accessed by chromium release assays. In the CT26 tumor model, DSP-0509 was administered at 5 mg/kg q1w intravenously. Radiation was dosed at 2 Gy/day for five sequential days from the starting day of DSP-0509 dosing. Splenocytes were collected 1 day after 3rd dose of DSP-0509 on day 21. In the chromium-release assay, CT26 was used as the target cell, and splenocytes from the control, DSP-0509, and combination groups were cultured in the presence of AH1 peptides known to be antigens of the CT26 cell line. We found that tumoricidal activity was significantly increased in the combination group compared with the control group at E/T ratios of 1:20, 1:40, and 1:80, respectively (Fig. 3A). Furthermore, we also found that the tumor volume in the respective tumor-bearing mice on day 20 was correlated with tumoricidal activity as assessed by chromium release (Fig. 3B). Splenic cells from mice after complete tumor elimination by combination therapy were cultured in the presence of AH1 peptide for 1 week, and IFNγ-producing cells were assessed by ELISPOT assays. As a result, without the addition of AH1 peptide, a few spots indicative of IFNγ production were observed in mice with complete tumor elimination, in addition the addition of AH1 significantly increased the number of spots (Fig. 3C). This data suggested that induction or expansion of CTLs that recognize tumor antigens is critical for complete tumor elimination by RT combined with DSP-0509. Next, to assess the establishment of long-term immunity, we re-implanted CT26 cells into the mice which eliminated first implantation by treatment with RT and DSP-0509. In the second implantation, which was conducted more than 90 days after the first implantation, we implanted CT26 cells at right dorsal flank as well as Renca cells at left dorsal flank. As a result, we found that both naïve mice and mice which eliminated first CT26 implantation accepted Renca cell grafts, but only the latter rejected second CT26 cell grafts (Fig. 3D). To verify that this rejection of rechallenged tumor was dependent on CD8^+^ T cells, we removed CD8^+^ cells by anti-CD8 antibody. It was confirmed that CD8^+^ T cells could be eliminated by administering 200 µg of anti-CD8 antibody three times every other day (Supplementary Fig. 4). Depletion of CD8^+^ T cells permitted the second CT26 engraftment in mice with complete tumor elimination by treatment with RT and DSP-0509, and the tumor growth was comparable to that in naïve mice (Fig. 3E). These results indicated that long-term anti-CT26 tumor immunity has been established in mice with complete tumor elimination by the combination of RT and DSP-0509. To verify the involvement of memory CD8^+^ T cells in the establishment of this long-term immunity, we analyzed peripheral blood samples from the control, RT, and combination groups by flow cytometry to evaluate the percentage of effector memory CD8^+^ T cells (CD8^+^CD62L^−^CD127^+^). The percentage of effector memory T cells was increased in the combination group compared with the control group (Fig. 3F). These data suggested that memory T cells are involved in the establishment of long-term memory immunity by the combination of RT and DSP-0509.

### A peripheral blood gene signature could predict the response to RT combined with DSP-0509

**Fig. 4:**
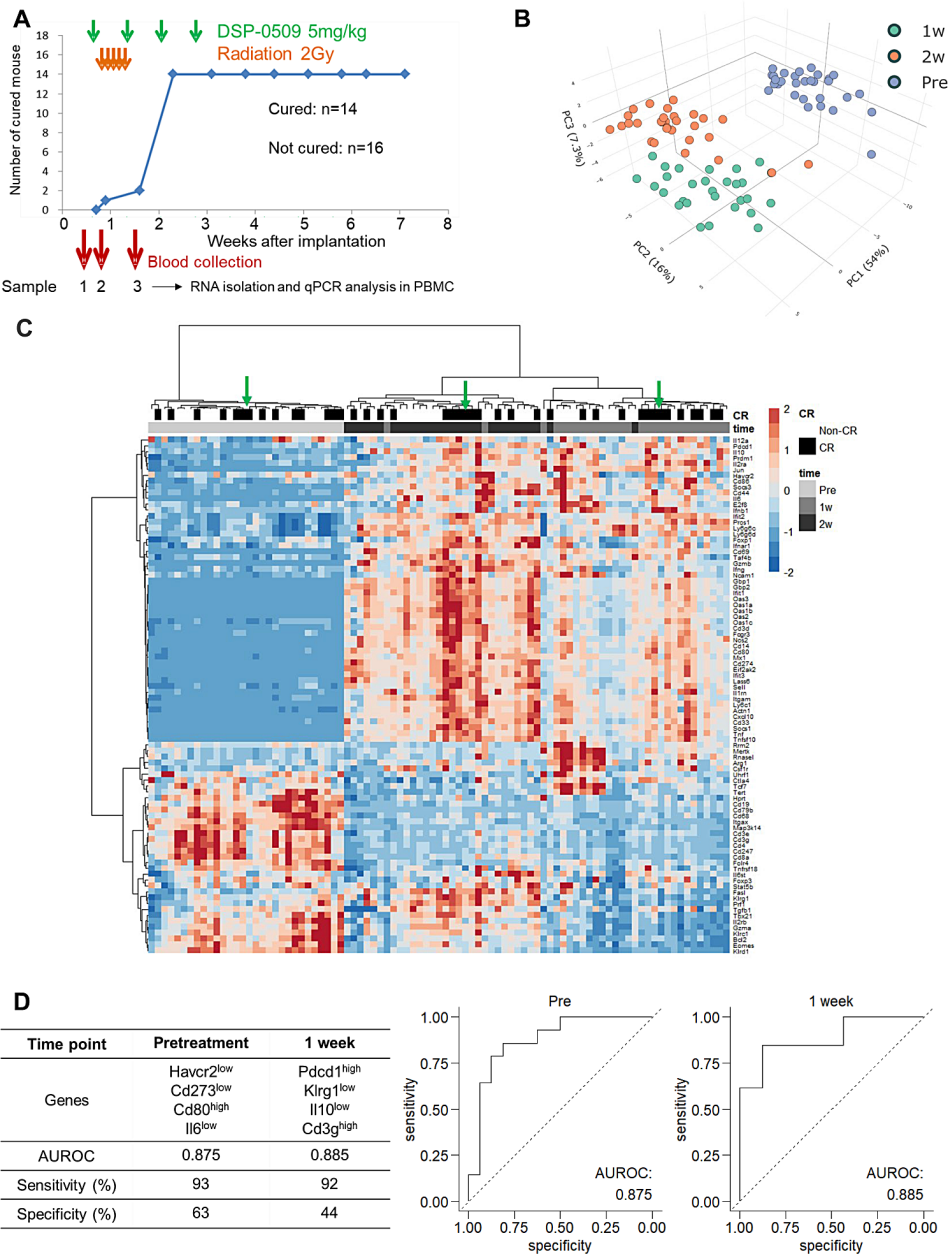
Peripheral blood gene signature can predict the response to the combination of RT with DSP-0509. **A.** Study schedule used for peripheral blood mRNA expression analysis in CT26 model mice treated with combined DSP-0509 and RT. **B**. Principal component analysis of the expression levels of 94 genes in PBLs. *n* = 30 at each time point. **C**. Hierarchical cluster analysis of the expression levels of 94 genes in PBLs. **D**. Multiple logistic regression analysis of the mRNA expression levels used to predict complete tumor regression after treatment with combined DSP-0509 and RT. Selected genes in and predictive performance of regression models are shown in the upper panel. Performance of each model is also shown by ROC curves in the bottom panel

Next, we hypothesized that the differences in the therapeutic response may be due to differences in the immune status of the tumor microenvironment. Thus, to explore whether there are any factors available to predict therapeutic response, blood was collected prior to treatment, 2 h after the first dose of DSP-0509 and 2 h after the second dose of DSP-0509. Then, gene expression analysis of immune-related 94 genes in blood cells was performed by qPCR (Fig. 4A). PCAs were performed based on gene expression profiles in PBLs. Interestingly, the three-dimensional plot showed that samples formed distinct clusters per time point (Fig. 4B). At this time, the two-dimensional plot of PC1 and PC2 clearly separated the pre- and post-treatment groups, but did not separate the first and second dose groups (Supplementary Fig. 5). On the other hand, PCAs did not reveal any separation between the mice that responded to treatment and mice that did not respond completely. We then used hierarchical clustering to determine whether a characteristic gene-expression profile exists in the mice with complete tumor elimination. Consequently, the time points at which PBLs were collected as well as the PCA data points were well separated into different cluster groups (Fig. 4C). Interestingly, there were partially enriched clusters of mice with complete tumor elimination among the clusters per time point (Fig. 4C). This led us to surmise that there is a characteristic expression profile in mice with complete tumor elimination. Thus, to identify the genes of PBLs that predict which mice respond to treatment, we performed logistic regression analysis with gene expression at each time point as the explanatory variables and the presence or absence of complete tumor elimination later as the objective variable. Consequently, among the expressed genes of PBLs at the pre-dose time point, we identified Havcr2 as a gene that could predict complete tumor elimination by treatment with combined DSP-0509 and RT with the highest predictive performance (Supplemental Table 1). Furthermore, among genes expressed at the 2 h post first dose of DSP-0509, Pdcd1 was found to predict complete tumor elimination with the highest predictive performance (Supplemental Table 1). Since it is possible to find prediction models with high predictive performance by combining multiple genes further, multiple logistic analysis was performed using the top eight genes with predictive relevance at each time point. Consequently, Havcr2^low^, Cd274^low^, Cd80^high^, and Il6^low^ combined had an AUROC of 0.875, which was higher than the AUROC of Havcr2 alone. This result suggested that combining multiple genes should be more accurate (Fig. 4D). Furthermore, using gene-expression of PBLs retrieved at 2 h after first dose of DSP-0509, we performed multiple logistic-regression analyses. It was found that the genes in PBLs from mice with complete tumor elimination were Cd274^high^, Klrg^low^, Il10^low^, and Cd3g^high^, and AUROC of the predictive model which combined these was 0.885. Pdcd1 was the gene with the highest prediction accuracy for a single gene, but the AUROC of this model was 0.75, revealing that combining more than one gene improved the prediction accuracy (Fig. 4D). These findings suggested that the gene expression in PBLs may be the biomarker for predicting complete tumor elimination when DSP-0509 is combined with RT.

### Gene expression related to anti-tumor immunity was increased in the tumor treated with combined RT and DSP-0509

**Fig. 5:**
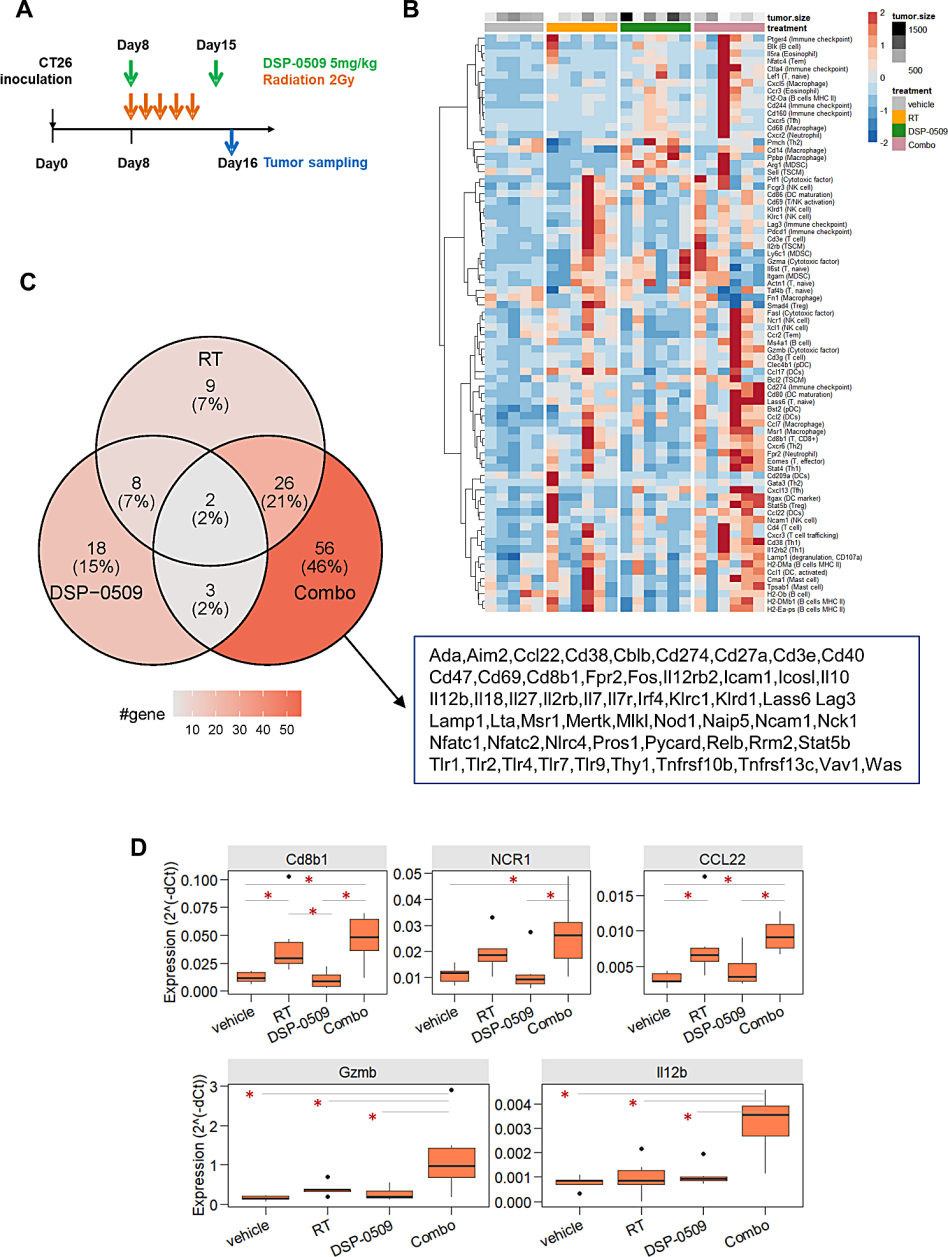
Gene expression related to anti-tumor immunity was increased in the tumor treated with combined RT and DSP-0509. **A.** Study schedule used for tumor mRNA expression analysis in CT26 model mice treated with combined DSP-0509 and RT. **B**. Hierarchical clustering analysis of mRNA expression data in each treatment group. **C**. Number of differentially expressed genes are shown in the Venn diagram. Each value shows the number of genes with increased expression more than 2 times in each treatment group compared to the control group. **D**. Expression levels of immune-related genes in each treatment group. *n* = 4–6 in each group. *False discovery rate (FDR)-adjusted *P* < 0.05 by the Student *t*-test

Next, we analyzed immune-related gene expression in tumors because the immune cells most involved in anti-tumor activity may be present in greater numbers in the tumor microenvironment. Tumor samples were collected 24 h after second dose of weekly DSP-0509 (Fig. 5A) in CT26 tumor-bearing mice model. Expressions of 218 immune related of genes were assessed by qPCR (Fig. 5B). Comparing gene expression by hierarchical clustering analysis revealed that expressions of various genes was elevated in the combination group (Fig. 5B). The overlap of the genes with elevated expression is shown in a Venn diagram (Fig. 5C). Twenty-six of the genes were elevated by the combination overlapped with those elevated by RT. Only three genes elevated by the combination were also elevated by DSP-0509 alone. In addition, focusing on individual genes, the expression of Cd8b1 in the tumor, which is considered a marker of CD8T cells, was significantly elevated by RT compared with the control group, and was further, although not significantly, elevated by the combination (Fig. 5D). The same tendency was observed for expression of Ccl22, which is considered a marker of DCs infiltration (Fig. 5D). DC related genes were consistently increased except for Cd209a (Supplementary Fig. 6). Expression of Ncr1, a marker of NK-cell infiltration, was significantly elevated only in the combination group relative to the control group (Fig. 5D). In addition, the expression of Gzmb, an anti-tumor effector molecule of immune cells with anti-tumor activity, was significantly elevated in the combination group compared with the control group only (Fig. 5D). This was also observed for Il12b expression (Fig. 5D). Treg and MDSC marker or related genes were tended to be increased in combination group (Supplementary Fig. 6). Thus, the combination of DSP-0509 and RT was found to induce anti-tumor immune cell markers as well as effector molecule in the tumor, leading to strong anti-tumor immunity.

## Discussion

In this study, enhancement of anti-tumor activity by DSP-0509 combined with RT was observed only at low but not high radiation doses (Supplementary Fig. 3). We think these findings suggest that a low dose of RT has optimal immune activating effects in combination with DSP-0509. It is reported that hypofractionated radiation (8 Gy x 3) is optimal for enhancing immunological activities such as MHC class I induction, cytokine induction, as well as not inducing immune suppressive mechanisms when RT is combined with ICBs including anti-CTLA-4 and anti-PD-1 antibodies [24, 25]. The radiation schedule used in this study (2 Gy x 5) is reported to induce immune suppressive cells in the tumor microenvironment [15], our data also showed gene expression related to MDSC was increased in RT group (Supplementary Fig. 6). But DSP-0509 enhanced tumor growth suppression in mice on this schedule of RT. Although TLR7 agonist is known to suppress MDSCs or Tregs [26, 27], DSP-0509 tended to increase these population in our study based on gene expression. To elucidate this discrepancy, we need to examine further studies in future by analyzing time course or flow cytometry analysis. Based on these data, it is considered that the optimal irradiation schedule operates by a different immune activation mechanism than even immunotherapy. The lack of concomitant efficacy at high doses in this combination with DSP-0509 may be attributed to excessive stimulation of myeloid cells including macrophages and DCs, because the immune activation mechanism mediated by each constituent of this therapy partially overlaps. When RT and DSP-0509 were combined, strong anti-tumor activity leading to complete tumor elimination was observed in the CT26 model. Also, additive tumor growth inhibition but not strong anti-tumor effects leading to complete tumor elimination was observed in the LM8 and 4T1 models. Previously, the microenvironment in the 4T1 model was reported to be immunosuppressive as well as low tumor mutation burden [28, 29], suggesting that achieving complete tumor elimination would be challenging in this model, but evaluating the ability of DSP-0509 to enhance the anti-tumor effect of RT was worthwhile. Since it is reported that anti-PD1 therapy is not effective in LM8 model [30], the tumor microenvironment of LM8 may be also considered immunosuppressive. It is likely that when compared to the combination of RT with chemotherapy, only the combination of DSP-0509 with RT can enhance the anti-tumor effect and prolong survival of tumor-bearing mice. We think that inducing long lasting anti-tumor immunity is one of the advantages of radioimmunotherapy. Immunotherapy differs from cytotoxic anti-cancer drug therapy, and differences in host immune status greatly affect the strength of the anti-tumor effects. Although strong anti-tumor effects leading to complete tumor elimination were observed in tumor-bearing mice treated with the combination of DSP-0509 and RT, complete tumor elimination was not observed in all tumor-bearing mice. Therefore, the present study explored factors affecting efficacy using gene-expression in peripheral immune cells. Although it is not clear from the present analysis which cells are expressing these genes, CD80 is known as an activation marker of monocytes in peripheral blood, suggesting that the activity of monocytes prior to tumor growth is a critical factor affecting the strength of anti-tumor activity induced by the combination with DSP-0509. Since monocytes are known to express the DSP-0509 target molecule TLR7, it is reasonable to attribute the strength of the anti-tumor activity of the combination with RT to strong DSP-0509-induced activation of monocytes via TLR7 signal upregulation. It was also shown that low IL-6 expression is required for the strong anti-tumor effect of DSP-0509 in the combination with RT, but may be the result of the anti-tumor effect since IL-6 acts negatively on the activation of CTLs [31]. In addition, lower expression of PD-L1 and TIM3 has been shown to be required for the strong anti-tumor effect of the combination, but TIM3 is a known marker of CTL exhaustion [32] and PD-L1 is a known ligand of PD-1 on CTLs [33], suggesting that a lower degree of CTL exhaustion is required for the strong anti-tumor effect of the combination treatment. Our findings suggested that DSP-0509 combined with RT resulted in increased level of memory T cells as well as the activation of CTLs that recognize cancer antigens in mice with complete tumor elimination, suggesting that it may be reasonable for the anti-tumor response of mice to the combination to differ according to expression of PD-L1 and TIM3, which are markers of CTL exhaustion. When we think of anti-tumor immunity, it is important to analyze immune cell alteration at the tumor site. Gene expression analysis in tumors suggested that DSP-0509 was primarily active in CD8T cells, particularly because of the elevation in expression of genes encoding effector molecules such as Gzmb and Il12. On the other hand, RT induced Cd8b1 gene expression, suggesting that it was the main reason for the increase in CD8^+^ T cell infiltration. In the combination group, Il7 was increased. IL-7 is reported to be important in memory T cell induction [34]. Thus, memory T cell induction by combination of RT and DSP-0509 can be explained by IL-7 induction. In this tumor analysis, DSP-0509 monotherapy did not increase Cd8a or Gzmb expression. In the previous study, expression of Cd8a and Gzmb was increased by DSP-0509 treatment [20], and this inconsistency can be explained by the difference of sampling points between studies. In this study, tumor samples were collected 1 day after the second dosing. Cd8a and Gzmb expressions were increased 4 days after dosing. Based on these data, it is suggested that combining of DSP-0509 and RT prolongs the duration of immune cell activation in the tumor microenvironment compared to DSP-0509 monotherapy.

## Conclusions

We clarified the combination of DSP-0509 and RT can increase anti-tumor effector molecules related to CD8^+^ T cells and myeloid cells in tumor. These resulted in potent anti-tumor activity that completely eliminates the tumor. We also found the gene signature in PBL to predict complete tumor elimination before combination treatment. We think our results strongly support combining the TLR7 agonist, DSP-0509, with RT, and future studies including clinical trials are expected.

### Electronic supplementary material

Below is the link to the electronic supplementary material.

Supplementary Material 1

Supplementary Material 2

Supplementary Material 3

Supplementary Material 4

## Data Availability

The datasets used or analyzed during the current study are available from the corresponding author on reasonable request.

## Abbreviations

AUROC: Area under receiver operating characteristic
CTL: Cytotoxic T lymphocyte
DC: Dendritic cell
HMGB1: High mobility group protein B1
IRF7: Interferon regulatory factor 7
ICB: Immune checkpoint blocker
MDSC: Myeloid derived suppressor cell
MYD88: Myeloid differentiation factor 88
NF-kB: Nuclear factor-kappa B
NK: Natural killer cell
PBL: Peripheral blood lymphocyte
PCA: Principal component analysis
RT: Radiation therapy
ROC: Receiver operating characteristic
TAM: Tumor associated macrophage
TLR7: Toll like receptor 7
TIL: Tumor infiltrated lymphocyte
Treg: Regulatory T cell

## References

[1] Aderem A, Ulevitch RJ. Toll-like receptors in the induction of the innate immune response. Nature. 2000;406(6797):782–7. 10963608 10.1038/35021228

[2] Medzhitov R, Janeway C. Jr. Innate immunity. N Engl J Med. 2000;343(5):338–44. 10922424 10.1056/NEJM200008033430506

[3] Barchet W, Wimmenauer V, Schlee M, Hartmann G. Accessing the therapeutic potential of immunostimulatory nucleic acids. Curr Opin Immunol. 2008;20(4):389–95. 18652893 10.1016/j.coi.2008.07.007

[4] Kobold S, Wiedemann G, Rothenfußer S, Endres S. Modes of action of TLR7 agonists in cancer therapy. Immunotherapy. 2014;6(10):1085–95. 25428647 10.2217/imt.14.75

[5] Swiecki M, Gilfillan S, Vermi W, Wang Y, Colonna M. Plasmacytoid dendritic cell ablation impacts early interferon responses and antiviral NK and CD8(+) T cell accrual. Immunity. 2010;33(6):955–66. 21130004 10.1016/j.immuni.2010.11.020 PMC3588567

[6] Le Mercier I, Poujol D, Sanlaville A, Sisirak V, Gobert M, Durand I, et al. Tumor promotion by intratumoral plasmacytoid dendritic cells is reversed by TLR7 ligand treatment. Cancer Res. 2013;73(15):4629–40. 23722543 10.1158/0008-5472.CAN-12-3058

[7] Drobits B, Holcmann M, Amberg N, Swiecki M, Grundtner R, Hammer M, et al. Imiquimod clears tumors in mice independent of adaptive immunity by converting pDCs into tumor-killing effector cells. J Clin Invest. 2012;122(2):575–85. 22251703 10.1172/JCI61034 PMC3266798

[8] Tyring S. Imiquimod applied topically: a novel immune response modifier. Skin Therapy Lett. 2001;6(6):1–4. 11298484

[9] Rook AH, Gelfand JM, Wysocka M, Troxel AB, Benoit B, Surber C, et al. Topical resiquimod can induce disease regression and enhance T-cell effector functions in cutaneous T-cell lymphoma. Blood. 2015;126(12):1452–61. 26228486 10.1182/blood-2015-02-630335 PMC4573868

[10] Brody JD, Ai WZ, Czerwinski DK, Torchia JA, Levy M, Advani RH, et al. In situ vaccination with a TLR9 agonist induces systemic lymphoma regression: a phase I/II study. J Clin Oncology: Official J Am Soc Clin Oncol. 2010;28(28):4324–32. 10.1200/JCO.2010.28.9793 PMC2954133 20697067

[11] Rolfo C, Giovannetti E, Martinez P, McCue S, Naing A. Applications and clinical trial landscape using toll-like receptor agonists to reduce the toll of cancer. 2023;7(1):26. 10.1038/s41698-023-00364-1 PMC9995514 36890302

[12] Baskar R, Yap SP, Chua KL, Itahana K. The diverse and complex roles of radiation on cancer treatment: therapeutic target and genome maintenance. Am J cancer Res. 2012;2(4):372–82. 22860229 PMC3410581

[13] Demaria S, Guha C, Schoenfeld J, Morris Z, Monjazeb A, Sikora A et al. Radiation dose and fraction in immunotherapy: one-size regimen does not fit all settings. so how does one Choose? 2021;9(4). 10.1136/jitc-2020-002038 PMC8031689 33827904

[14] Liu T, Pei P, Shen W, Hu L, Yang K. Radiation-Induced Immunogenic Cell Death Cancer Radioimmunotherapy. 2023:e2201401. 10.1002/smtd.202201401 36811166

[15] Colciago RR, Fischetti I, Giandini C, La Rocca E, Rancati TT, Rejas Mateo A et al. Overview of the synergistic use of radiotherapy and immunotherapy in cancer treatment: current challenges and scopes of improvement. 2023;23(2):135–45. 10.1080/14737140.2023.2173175 36803369

[16] Xu J, Escamilla J, Mok S, David J, Priceman S, West B, et al. CSF1R signaling blockade stanches tumor-infiltrating myeloid cells and improves the efficacy of radiotherapy in prostate cancer. Cancer Res. 2013;73(9):2782–94. 23418320 10.1158/0008-5472.CAN-12-3981 PMC4097014

[17] Liang H, Deng L, Hou Y, Meng X, Huang X, Rao E et al. Host STING-dependent MDSC mobilization drives extrinsic radiation resistance. 2017;8(1):1736. 10.1038/s41467-017-01566-5 PMC5701019 29170400

[18] Kachikwu EL, Iwamoto KS, Liao YP, DeMarco JJ, Agazaryan N, Economou JS, et al. Radiation enhances regulatory T cell representation. Int J Radiat Oncol Biol Phys. 2011;81(4):1128–35. 21093169 10.1016/j.ijrobp.2010.09.034 PMC3117954

[19] Zhang Z, Liu X, Chen D, Yu J. Radiotherapy combined with immunotherapy: the dawn of cancer treatment. Signal Transduct Target Therapy. 2022;7(1):258. 10.1038/s41392-022-01102-y PMC9338328 35906199

[20] Ota Y, Nagai Y, Hirose Y, Hori S, Koga-Yamakawa E, Eguchi K, et al. DSP-0509, a systemically available TLR7 agonist, exhibits combination effect with immune checkpoint blockade by activating anti-tumor immune effects. Front Immunol. 2023;14:1055671. 36793737 10.3389/fimmu.2023.1055671 PMC9922899

[21] Dovedi SJ, Adlard AL, Ota Y, Murata M, Sugaru E, Koga-Yamakawa E, et al. Intravenous administration of the selective toll-like receptor 7 agonist DSR-29133 leads to anti-tumor efficacy in murine solid tumor models which can be potentiated by combination with fractionated radiotherapy. Oncotarget. 2016;7(13):17035–46. 26959743 10.18632/oncotarget.7928 PMC4941369

[22] Adlard AL, Dovedi SJ, Telfer BA, Koga-Yamakawa E, Pollard C, Honeychurch J, et al. A novel systemically administered toll-like receptor 7 agonist potentiates the effect of ionizing radiation in murine solid tumor models. Int J Cancer. 2014;135(4):820–9. 24390981 10.1002/ijc.28711 PMC4286010

[23] Koga-Yamakawa E, Murata M, Dovedi SJ, Wilkinson RW, Ota Y, Umehara H, et al. TLR7 tolerance is independent of the type I IFN pathway and leads to loss of anti-tumor efficacy in mice. Cancer Immunol Immunotherapy: CII. 2015;64(10):1229–39. 10.1007/s00262-015-1730-4 PMC11029383 26091797

[24] Arina A, Gutiontov SI. Radiotherapy and Immunotherapy for Cancer: from systemic to Multisite. 2020;26(12):2777–82. 10.1158/1078-0432.CCR-19-2034 PMC10759929 32047000

[25] Kwon ED, Drake CG, Scher HI, Fizazi K, Bossi A, van den Eertwegh AJ, et al. Ipilimumab versus placebo after radiotherapy in patients with metastatic castration-resistant prostate cancer that had progressed after docetaxel chemotherapy (CA184-043): a multicentre, randomised, double-blind, phase 3 trial. Lancet Oncol. 2014;15(7):700–12. 24831977 10.1016/S1470-2045(14)70189-5 PMC4418935

[26] Spinetti T, Spagnuolo L, Mottas I, Secondini C, Treinies M, Rüegg C, et al. TLR7-based cancer immunotherapy decreases intratumoral myeloid-derived suppressor cells and blocks their immunosuppressive function. Oncoimmunology. 2016;5(11):e1230578. 27999739 10.1080/2162402X.2016.1230578 PMC5139641

[27] Wang C, Zhou Q, Wang X, Wu X, Chen X, Li J, et al. The TLR7 agonist induces tumor regression both by promoting CD4⁺T cells proliferation and by reversing T regulatory cell-mediated suppression via dendritic cells. Oncotarget. 2015;6(3):1779–89. 25593198 10.18632/oncotarget.2757 PMC4359331

[28] Mosely SI, Prime JE, Sainson RC, Koopmann JO, Wang DY, Greenawalt DM, et al. Rational selection of Syngeneic Preclinical Tumor models for Immunotherapeutic Drug Discovery. Cancer Immunol Res. 2017;5(1):29–41. 27923825 10.1158/2326-6066.CIR-16-0114

[29] Schrörs B, Boegel S, Albrecht C, Bukur T, Bukur V, Holtsträter C, et al. Multi-omics characterization of the 4T1 murine mammary gland Tumor Model. Front Oncol. 2020;10:1195. 32793490 10.3389/fonc.2020.01195 PMC7390911

[30] Katsuki S, Takahashi Y. Radiation therapy enhances systemic antitumor efficacy in PD-L1 therapy regardless of sequence of radiation in murine osteosarcoma. 2022;17(7):e0271205. 10.1371/journal.pone.0271205 PMC9273087 35816501

[31] Tsukamoto H, Fujieda K, Senju S, Ikeda T, Oshiumi H, Nishimura Y. Immune-suppressive effects of interleukin-6 on T-cell-mediated anti-tumor immunity. Cancer Sci. 2018;109(3):523–30. 29090850 10.1111/cas.13433 PMC5834784

[32] Wolf Y, Anderson AC, Kuchroo VK. TIM3 comes of age as an inhibitory receptor. Nat Rev Immunol. 2020;20(3):173–85. 31676858 10.1038/s41577-019-0224-6 PMC7327798

[33] Sharpe AH, Pauken KE. The diverse functions of the PD1 inhibitory pathway. Nat Rev Immunol. 2018;18(3):153–67. 28990585 10.1038/nri.2017.108

[34] Cieri N, Camisa B, Cocchiarella F, Forcato M, Oliveira G, Provasi E, et al. IL-7 and IL-15 instruct the generation of human memory stem T cells from naive precursors. Blood. 2013;121(4):573–84. 23160470 10.1182/blood-2012-05-431718

