# Supplementary material for: TLR7 agonist, DSP-0509, with radiation combination therapy enhances anti-tumor activity and modulates T cell dependent immune activation
Source: BMC Immunol. 2024 Jul 25;25:48. doi: 10.1186/s12865-024-00643-x (PMC11270965; doi:10.1186/s12865-024-00643-x)
Supplement: Supplementary file 4 — Supplementary Material 4 [file 12865_2024_643_MOESM4_ESM.docx]

**Supplemental materials**


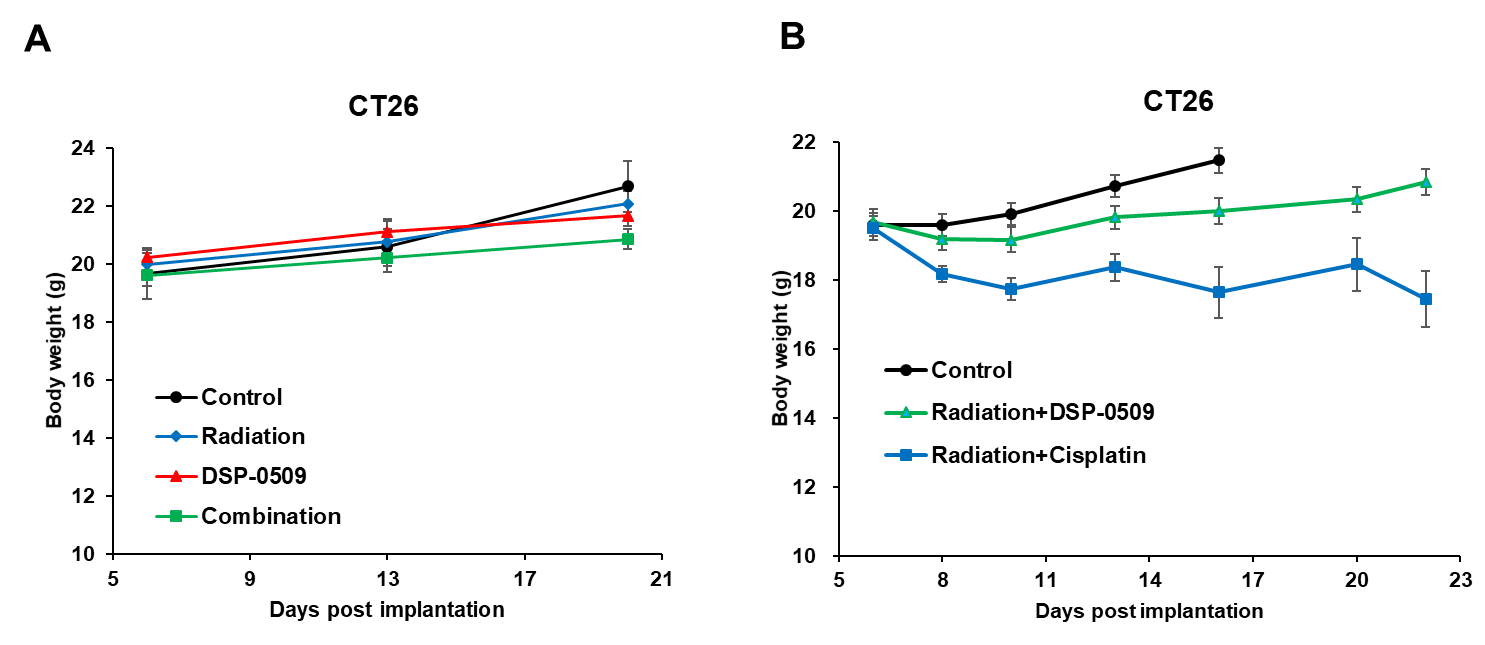


**Supplementary Figure. 1** **A.** Body weight transition in Fig. 1B. Values are the average ± S.E.M for each group. **B.** Body weight transition in Fig. 1C. Values are the average ± S.E.M for each group.


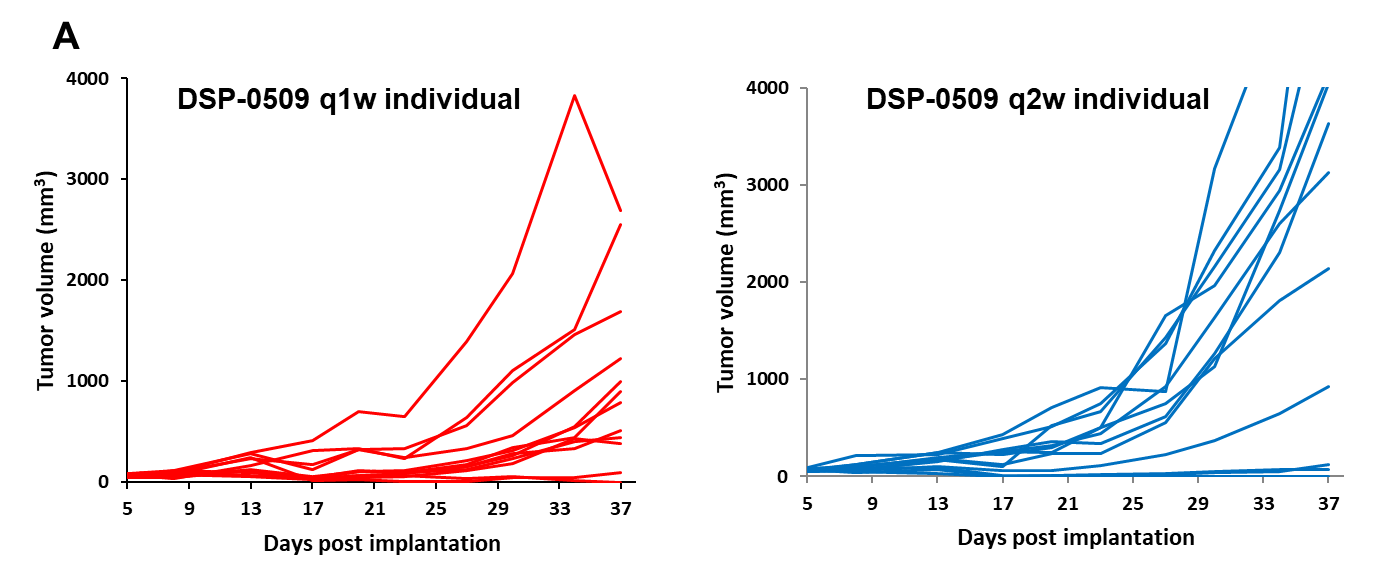


**Supplementary Figure. 2** **A.** Individual tumor growth data in Fig. 2A


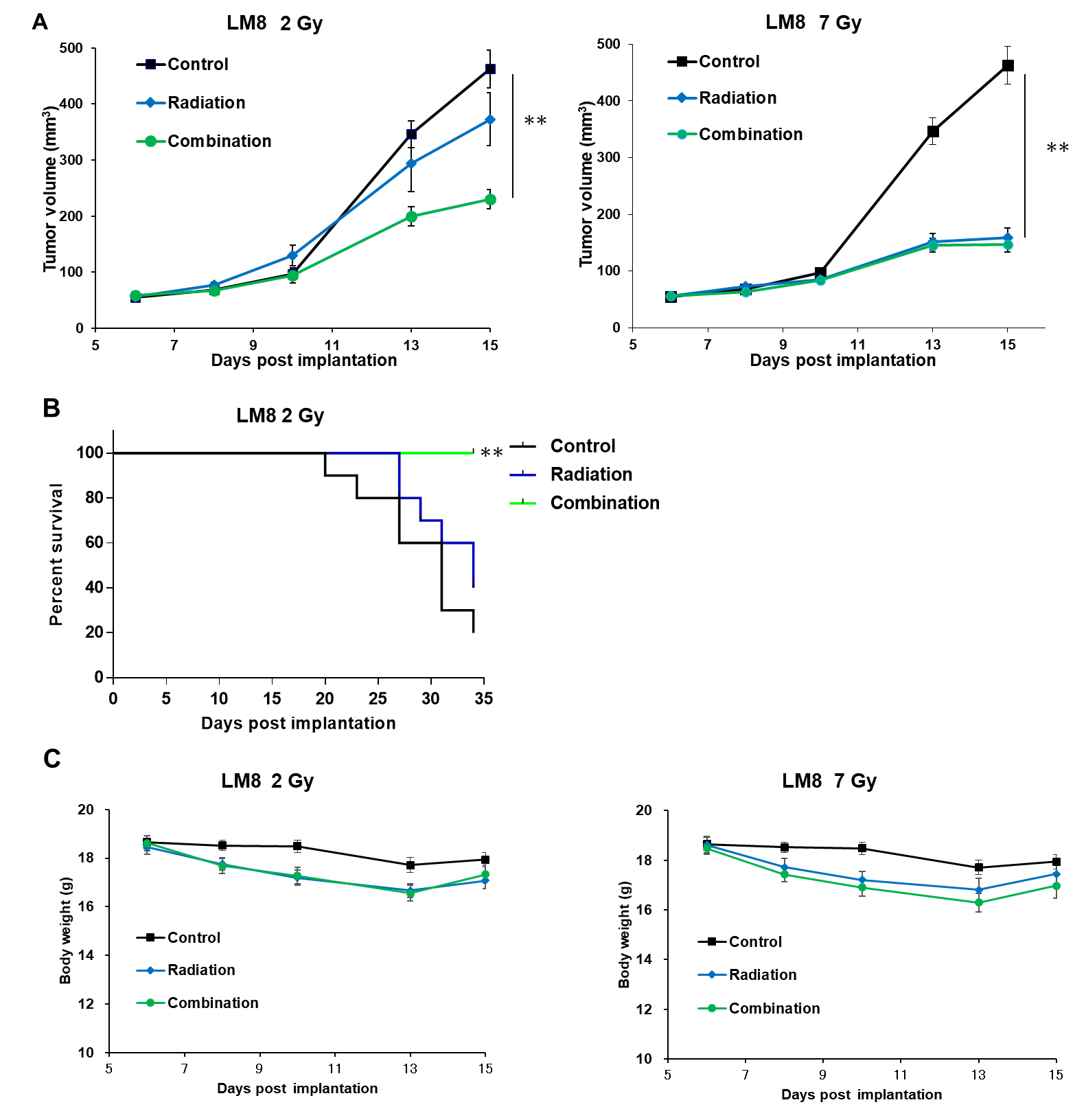


**Supplementary Figure. 3** Enhanced anti-tumor activity was dependent on radiation dose. **A.** Combination of radiation with DSP-0509 in LM8 model. DSP-0509 was administered at 5 mg/kg i.v. once a week interval. Dosing of radiation was conducted 5 sequential days from the day of first dosing of DSP-0509. **P < 0.01 vs control by Tukey test . n = 10 / each group. **B.** Kaplan-Meier curve in LM8 model. Radiation of 2 Gy was dosed 5 sequential days from the from the day of first dosing of DSP-0509. **P < 0.01 vs control by log rank test. n = 10 / each group. **C.** Body weight transition in supplementary Fig. 3A.


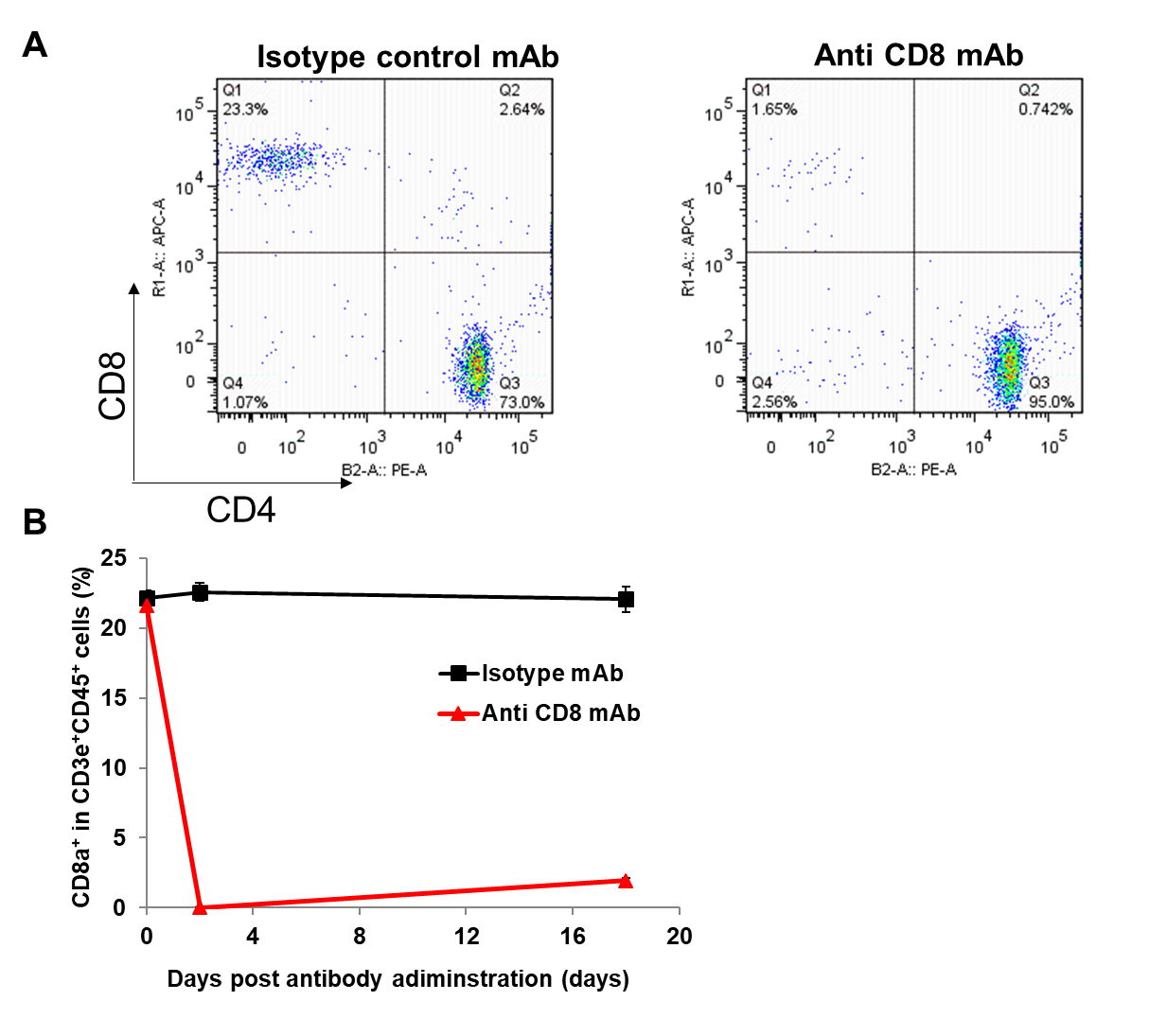


**Supplementary Figure. 4** Confirmation of CD8^+^ T cell depletion by anti-CD8mab. **A.** Representative plot of CD8 in CD45^+^CD3e^+^in depleted mouse. **B.** Time course of CD8^+^ T cell population after antibody administration.


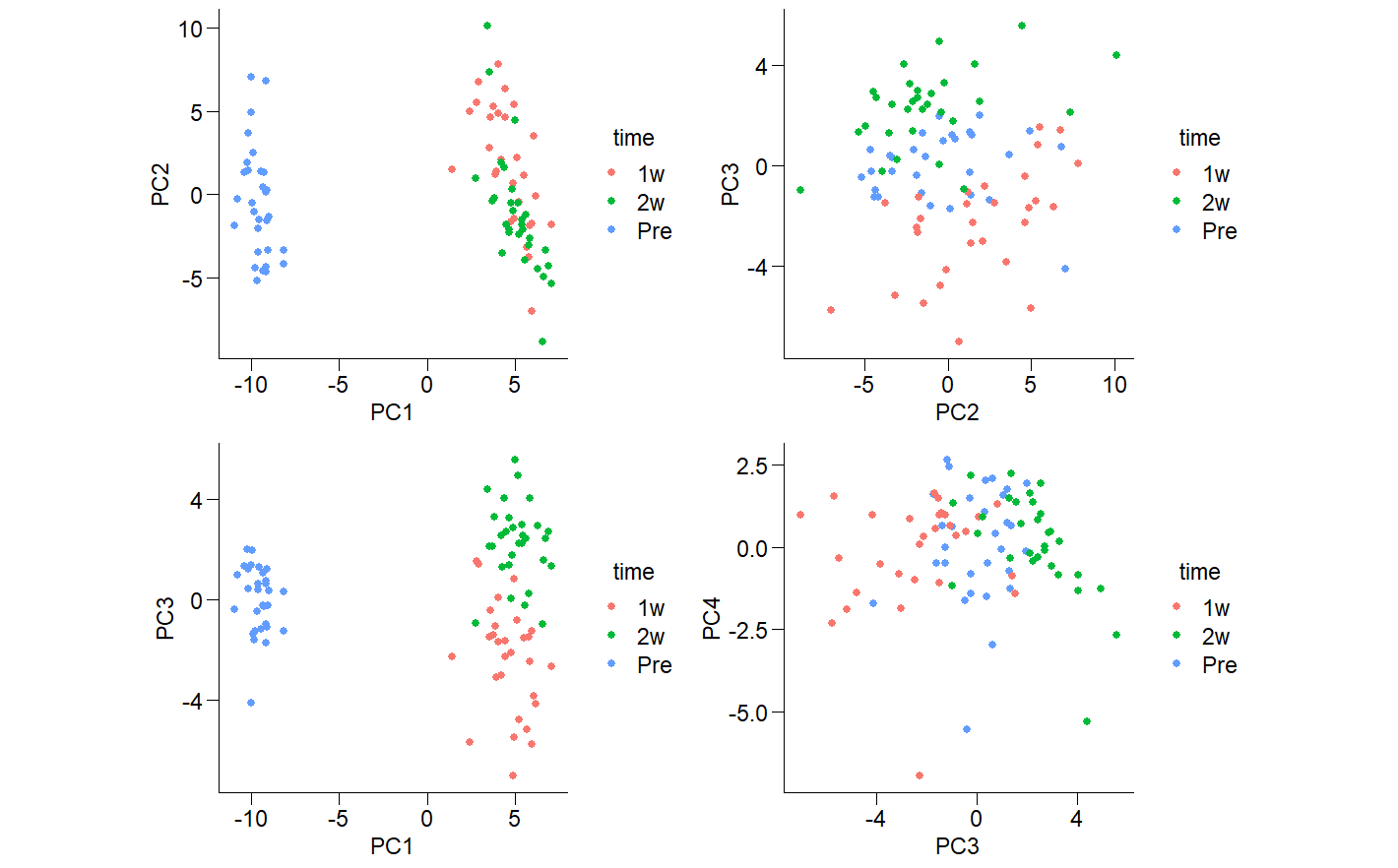


**Supplementary Figure. 5** Principle component analysis using the expression of 94 immune related genes from each timepoints.


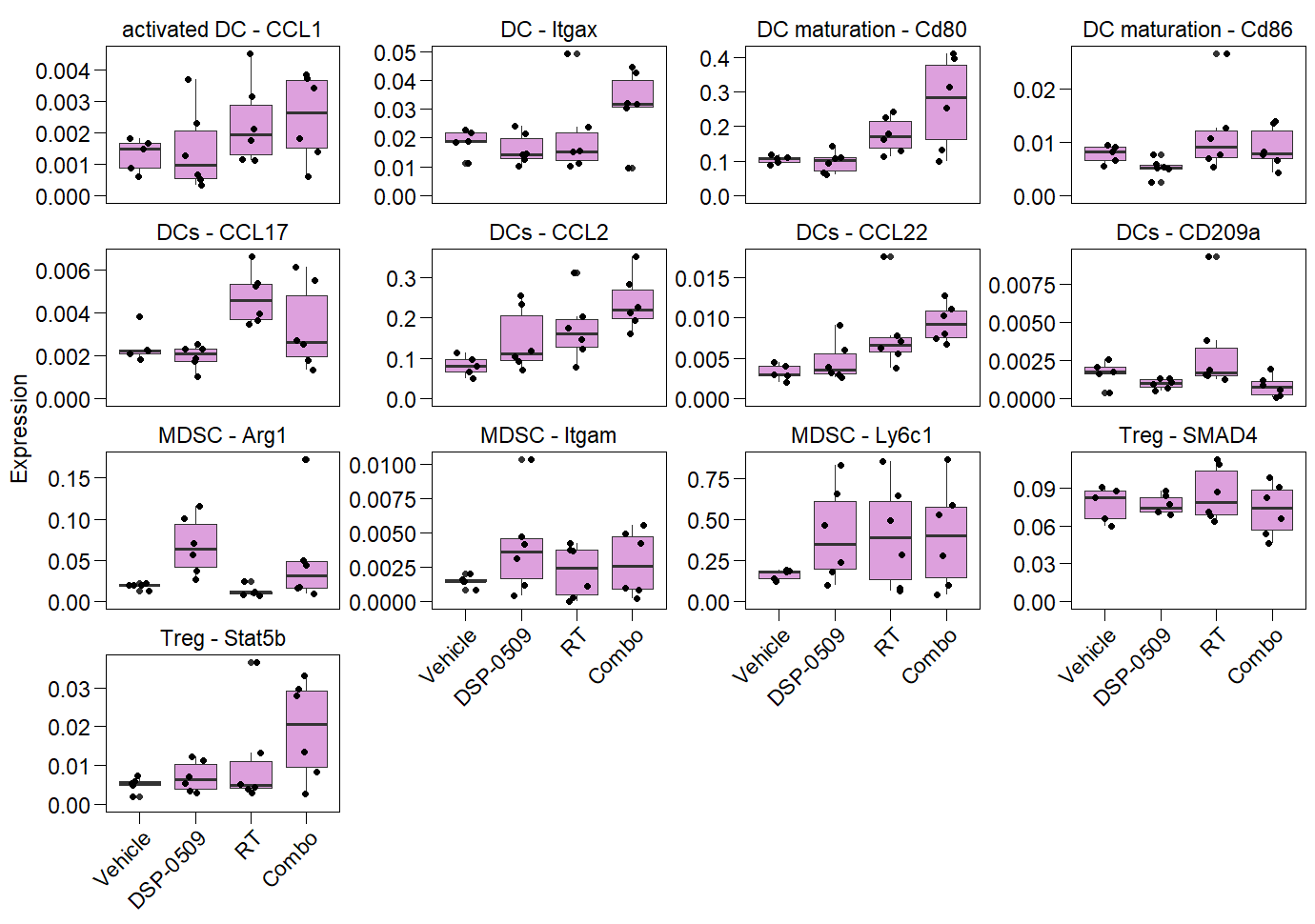


**Supplementary Figure. 6** Individual gene expression in CT26 tumor related to Treg, MDSC and DC.


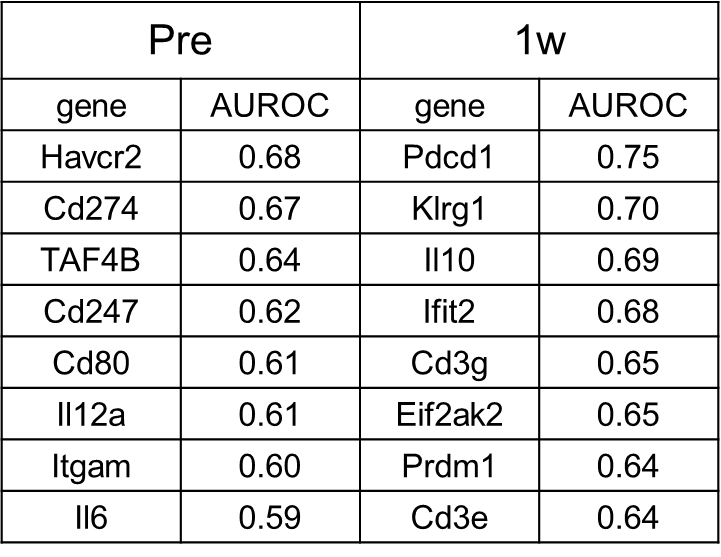


**Supplementary Table. 1** Top 8 genes of high performance to predict complete tumor regression based on logistic regression analysis.

**Supplementary Table. 2** Primer list for qPCR

**Supplementary Table. 3** PCR raw data
